# 11*β*-Hydroxysteroid Dehydrogenase 2 in Preeclampsia

**DOI:** 10.1155/2016/5279462

**Published:** 2016-04-21

**Authors:** Katarzyna Kosicka, Anna Siemiątkowska, Franciszek K. Główka

**Affiliations:** Department of Physical Pharmacy and Pharmacokinetics, Poznan University of Medical Sciences, 6 Święcickiego Street, 60-781 Poznań, Poland

## Abstract

Preeclampsia is a serious medical problem affecting the mother and her child and influences their health not only during the pregnancy, but also many years after. Although preeclampsia is a subject of many research projects, the etiology of the condition remains unclear. One of the hypotheses related to the etiology of preeclampsia is the deficiency in placental 11*β*-hydroxysteroid dehydrogenase 2 (11*β*-HSD2), the enzyme which in normal pregnancy protects the fetus from the excess of maternal cortisol. The reduced activity of the enzyme was observed in placentas from pregnancies complicated with preeclampsia. That suggests the overexposure of the developing child to maternal cortisol, which in high levels exerts proapoptotic effects and reduces fetal growth. The fetal growth restriction due to the diminished placental 11*β*-HSD2 function may be supported by the fact that preeclampsia is often accompanied with fetal hypotrophy. The causes of the reduced function of 11*β*-HSD2 in placental tissue are still discussed. This paper summarizes the phenomena that may affect the activity of the enzyme at various steps on the way from the gene to the protein.

## 1. Introduction

Preeclampsia (PE) is a particular type of hypertension that is recognized only in pregnant women. PE is usually diagnosed after 20 weeks of gestation, but occasionally it can be recognized in the first 48 hours after delivery. The diagnostics is based on the elevated arterial blood pressure (diastolic and systolic blood pressure: ≥140 mmHg and ≥90 mmHg, resp.), accompanied by the significant loss of protein in the urine (≥300 mg/24 h). PE is a pregnancy related disorder, so it disappears within the 6 months after delivery [1]. The great clinical relevance of PE is evidenced by the fact that it affects the health and life of both the mother and her child. PE increases the risk of clotting disorders, renal dysfunction, or death of a pregnant woman. It is associated with the higher percentage of the premature births or stillbirths. Importantly, PE promotes also the intrauterine growth restriction (IUGR), thus giving birth to a child that is too small for its gestational age and gender [2, 3]. Low birth weight in turn increases the baby's predisposition to cardiovascular diseases as well as metabolic and psychiatric disorders in adulthood. This phenomenon is explained by the theory of “fetal programming,” introduced by Barker, that describes the impact of prenatal environment on the development of serious diseases in adult life [4, 5].

The abnormalities associated with PE have attracted researchers' attention for many years. The understanding of the precise mechanism of PE development could possibly help in the early diagnosis, prevention, and treatment of this disorder, but unfortunately the causes of this specific form of hypertension have not been yet fully explained [6, 7]. It is suggested that, among many phenomena associated with PE, there is a decreased activity of 11*β*-hydroxysteroid dehydrogenase 2 (11*β*-HSD2). This enzyme is crucial for cortisol (F) metabolism in placenta, thus maintaining a concentration gradient between the F levels in the mother's and the child's compartments [8]. Low birth weight in babies born from preeclamptic pregnancies could be then a consequence of an excess of F in the uterus [4, 8], as in the case of the apparent mineralocorticoid excess (AME) syndrome—an inherited, rare form of hypertension that is caused by a missense mutation in the* HSD11B2* gene encoding 11*β*-HSD2 [9]. The levels of circulating F in the mother are significantly different, approximately 10 times higher than those observed in the fetus. Therefore, even a small change in the activity of the placental 11*β*-HSD2 can result in a substantial increase of the amount of F that reaches the fetus [4, 5].

The paper summarizes the current literature concerning the placental 11*β*-HSD2 with a particular emphasis on the enzyme activity in pregnant women suffering from PE. It also discusses the mechanisms involved in the enzyme activity regulation that hypothetically may be responsible for the altered function of the protein over the course of PE.

## 2. 11***β***-Hydroxysteroid Dehydrogenase Type 2

**Figure 1:**
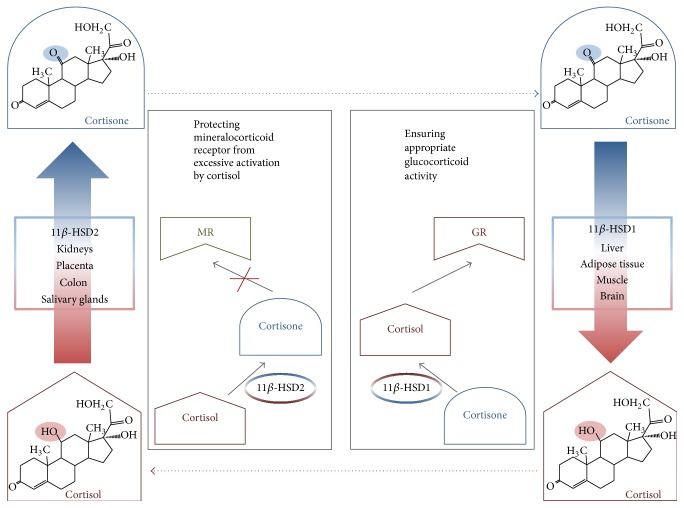
The function of human 11*β*-hydroxysteroid dehydrogenases. Cortisol is metabolized in the aldosterone target tissues (e.g., kidneys, colon, and salivary glands) to cortisone by 11*β*-HSD2. This enzymatic inactivation is a prereceptor mechanism that ensures the selectivity of mineralocorticoid receptor to aldosterone. Placenta cannot be considered as mineralocorticoid target tissue, and although the reaction of cortisol inactivation catalyzed by placental 11*β*-HSD2 is the same as in kidneys, the role of the process is different—it protects the developing fetus from the excess of maternal cortisol. 11*β*-HSD1 is expressed in glucocorticoid target tissues (e.g., liver, adipose tissue, muscle, and brain) where it ensures the cortisol concentration maintaining the proper activation of glucocorticoid receptor.

11*β*-HSD2 catalyzes the conversion of F to the biologically inactive cortisone (E) and plays a key role in the prereceptor metabolism of endogenous glucocorticoids (GCs). The protein is localized in the aldosterone target tissues, characterized by the abundant expression of the mineralocorticoid receptor (MR), so mainly in distal renal tubules, colon, and salivary glands. In the kidney, 11*β*-HSD2 is primarily responsible for the selectivity of the MR for aldosterone while in the other tissues it controls the availability of F and protects the glucocorticoid receptor (the receptor which is basic for GC action) from the excessive activation (Figure 1) [8–10].

In pregnancy, there is an additional source of 11*β*-HSD2—the placenta—a tissue with the most abundant expression of this enzyme [10, 11]. This expression is induced early during the trophoblast differentiation (syncytialization) and is blocked under the hypoxic condition [12]. The highest expression and amount of the placental 11*β*-HSD2 are observed in the syncytiotrophoblast while it is practically not detected in the fetal membranes [13–15]. The first record about the activity of 11*β*-HSD in the placenta appeared in 1960 thanks to Osinski et al., but researchers began to explore its properties in detail mostly in the 1980s. In 1993 Seckl et al. made the first isolation of the protein from the placenta. Later, after two isoforms, 11*β*-HSD1 and 11*β*-HSD2, had been distinguished, it was also confirmed that the placental isoform is the same type as the one discovered previously in kidneys, and so 11*β*-HSD2 [10, 16].

The placental 11*β*-HSD2 controls the overall availability of GCs in the tissue and is involved in the regulation of water and electrolyte transport across the placenta. Notably, this placental enzyme provides an additional function. It constitutes a specific barrier protecting the fetus from the maternal GCs, whose concentrations in pregnant woman are several times higher than those observed in the developing fetus [8, 14, 15]. Undoubtfully, the role of the placental 11*β*-HSD2 is pivotal, because F, essential in certain quantities to the proper development of fetal organs (e.g., the production of surfactant and maturation of the lungs, brain, and other organs), has a detrimental proapoptotic effect when it is in excess [4, 5]. Notably, the literature data point to a very high variability in the placental 11*β*-HSD2 activity between pregnant women. The fetuses may be then exposed to a substantially different amount of GCs in the uterus [11, 17].

Pregnancy is a physiological transient state of hypercortisolism. As a result of the activation of the hypothalamic-pituitary-adrenal axis, the plasma F levels increase progressively with gestation, reaching in the third trimester the concentrations that are several times higher than those observed before pregnancy [4, 18]. An increase in the expression and activity of the placental 11*β*-HSD2 with gestation can be observed, providing an effective protection of the child from the maternal GCs, which are lipophilic in their chemical nature and, therefore, can freely pass through the placenta [5, 11, 19]. The trend towards increasing activity of the placental 11*β*-HSD2 with gestation is not maintained in the last two weeks of pregnancy, when the activity of the enzyme decreases significantly, probably due to the mechanisms involved in the labour [20]. Interestingly, the decline in the activity is not reflected in the reduced* HSD11B2* expression [19, 20]. The mode of delivery affects neither the mRNA level nor the 11*β*-HSD2 protein activity [11, 13, 20], although F concentrations in the cord blood are higher in the case of the spontaneous labour [20, 21]. Expression and activity of the enzyme do not depend on the infant's gender [22, 23].

A significant negative correlation was described between the activity of placental 11*β*-HSD2 and the F/E ratio assessed in the umbilical venous blood [11]. Moreover, Shams et al. reported that F levels were much lower, while E levels were much higher in the cord blood than in the maternal circulation [11], confirming an important role of the placental 11*β*-HSD2 in the inactivation of F. Notably, the placental 11*β*-HSD2 is not an impermeable barrier for GCs; approximately 25% of F circulating in the fetus at term is of maternal origin [24]. Furthermore, despite the abundant expression of 11*β*-HSD2 in fetal tissues, the developing child lacks isoform 1 of the enzyme (11*β*-HSD1) until late gestation, when 11*β*-HSD1 is expressed in tissues whose maturation is glucocorticoid-dependent (e.g., lungs and liver). It is because the function of 11*β*-HSD1 is opposite to 11*β*-HSD2 and involves the regeneration of F from E. The low activity (or the lack of activity) of 11*β*-HSD1 in the fetus in early and mid-gestation prevents from F regeneration and thereby helps to avoid the increasing F levels in the developing organism [10].

## 3. Function of Placental 11***β***-HSD2 in PE

The activity of placental 11*β*-HSD2 in PE is reduced, which results in the increased F levels in the venous cord blood of the pregnant patients [25]. Moreover, F, which is practically not detected in the placentas of uncomplicated pregnancies, is present in almost 80% of PE cases [26]. These two facts clearly suggest the impaired conversion of F to E in the disease. Both the reduced activity of placental 11*β*-HSD2 [25] and the higher F levels in the placenta [26] exert a negative effect on infant's birth weight [25, 26], which may indicate a role of the impaired F metabolism in the pathogenesis of PE.

Interestingly, the activity of placental 11*β*-HSD2 in the pregnancies with high risk of PE is increased until the 10th week of gestation. It suggests that initially the F metabolism is more intensive in this specific type of hypertension. Unfortunately, it has been not clarified whether the disturbances in the GC metabolism underlie the PE development or they are a response to the irregularities arising from this disease [27].

## 4. How Can the Function of 11***β***-HSD2 Be Disturbed? Possible Explanations

There are several hypotheses concerning the mechanisms that may lead to the diminished function of 11*β*-HSD2. The next part of this paper describes those mechanisms and discusses the literature data supporting the particular hypotheses. It has to be emphasized though that the literature on placental 11*β*-HSD2 is descriptive, showing mostly associations, and very little is known about the role of placental 11*β*-HSD2 in the pathogenesis of PE.

### 4.1. Variations in the Sequence of* HSD11B2* Gene

Alterations in the DNA sequence of the coding region of* HSD11B2* gene have to be considered as the mechanism that can explain the reduced activity of 11*β*-HSD2. As mentioned above, this phenomenon has been confirmed to be the basis of AME. The* HSD11B2* gene, which is approximately 6200 kb long and consists of five exons, is located on chromosome 16, locus 16q22 [9, 10]. Missense mutation in the coding region of* HSD11B2* results in a different amino acid composition of the protein, which in turn may cause the partial or total reduction of the catalytic activity of the enzyme. However, the polymorphisms in noncoding regions of the gene must not be omitted when considering the* HSD11B2* sequence variations that influence the 11*β*-HSD2 function. There are reports of a major role of sequence variations within untranslated regions (UTRs) in the development of various diseases [28]. Therefore, the sequences of 5′-UTR and 3′-UTR have to be analyzed because of the regulatory role in gene expression that the UTRs play. Moreover, the promoter region of the gene is crucial for the initiation of transcription. There are reports describing the mutations in the promoter region of* HSD11B2*: G-209A and G126A that resulted in the lower affinity of Sp1 and NF1 transcriptional factors and consequently in the reduced expression of* HSD11B2* in salt-sensitivity patients [29]. Furthermore, a relationship between the microsatellite polymorphism [CA] (tandem repeats of CA nucleotides) in fetal intron 1 of* HSD11B2* and maternal plasma F levels was indicated [30].

It has been suggested that PE may be inherited because of the fact that women with the familial history of PE (mothers and sisters of those women suffered from PE) are more susceptible to its development. More than 60 genes candidates have been proposed so far as potentially involved in the disease development [31]. The published results did not confirm the relationship between the polymorphisms within* HSD11B2* and PE, but it has to be emphasized that only one study is available in this field. In the mentioned case-control study, Shimodaira et al. did not analyze the whole coding sequence of* HSD11B2* but focused on three selected single nucleotide polymorphisms (SNPs) within the gene [32]. The authors did not find evidence for the significant association between polymorphic variants of the* HSD11B2* gene and PE. However, the conclusions stated by Shimodaira et al. did not exclude entirely a relationship between the alterations in the gene sequence and development or severity of PE. It is emphasized that presence of modifying environmental or genetic factors should be considered when searching for the causes of the decreased activity of 11*β*-HSD2.

### 4.2. Reduced Placental 11*β*-HSD2 mRNA Levels

In theory, when the structure of a gene is correct, an observed impaired activity of the enzyme could be due to the lower gene expression in the particular tissue, reflected by a smaller amount of mRNA. This, in turn, results in a reduced level of the protein produced. The studies show that despite the similar expression of placental* HSD11B2* in the first trimester of pregnancies at both low and high PE risk [27], the mRNA level of* HSD11B2* measured at term is up to three times lower in patients who finally developed PE [33, 34]. Simultaneously, the decreased expression of 15-hydroxyprostaglandin dehydrogenase is observed, probably as a consequence of the excess of F [33].

Epigenetic modifications are very important when considering the potential causes of the reduced mRNA levels. They do not disturb DNA sequence but can significantly affect the transcription and translation efficiency and are more and more often identified as a link between the adverse prenatal environment and the health problems in adult life. DNA methylation is one of the most significant epigenetic modifications in mammals, involving the binding of methyl group to the 5′ position of a cytosine, usually in the GC dinucleotide sequence [35, 36]. Two GC-rich clusters, so-called “CpG islands,” have been localized in the human promoter and exon 1 of* HSD11B2* (nucleotides −633 to −97 and −77 to +460) and the next two were confirmed in exon 5 and the downstream region (nucleotides +5,569 to +5,721 and +7,357 to +7,515). Those regions of the gene are most susceptible to the methylation process. Furthermore, the* in vitro* studies have shown that hypermethylation within the promoter of the* HSD11B2* was associated with significantly reduced expression of the gene [37]. Therefore, it can be suspected that the hypermethylation in* HSD11B2* might be responsible for transcriptional limitation. With regard to the placental form of the enzyme, the phenomenon was confirmed* in vivo* in both the rat model [38] and the study concerning pregnant women [39, 40]. Interestingly, some authors pointed out that the correlation between the methylation and the expression of the placental* HSD11B2* refers only to the female fetuses [41]. The mechanism by which methylation influences the expression of* HSD11B2* is complex and involves the impeded binding of the transcription factors (e.g., NF1, Sp1/Sp3, and NF-*κ*B) to the specific DNA sequences as well as the easier binding of the particular DNA repressors [36, 37].

Changes in the methylation level within the genome were identified as one of the potential causes of arterial hypertension in nonpregnant population [35]. The hypermethylation of the specific CpG sites of* HSD11B2* has been detected in the placentas of women with IUGR [40] and in the fetal kidney in the rat model reflecting human IUGR [36]. Attempts have been made to explain the reduced* HSD11B2* expression in the placental tissue by the analysis of the methylation levels within the* HSD11B2* gene in the placentas from pregnancies complicated by PE. The results obtained so far have not confirmed that the higher DNA methylation level is associated with the decreased activity of placental 11*β*-HSD2 [42], even when the significantly diminished expression of both mRNA and protein levels was observed [34]. Moreover, the hypomethylation of the discussed gene detected in the cord blood of cases with PE was reported [43], suggesting the increased expression of 11*β*-HSD2. However, it is difficult to conclude whether the hypomethylation determined in cord blood reflects the activity of placental 11*β*-HSD2 because of the tissue-specific nature of methylation. Additionally, the preliminary studies indicated that* HSD11B2* methylation level quantified in the cord blood did not correlate with the one measured in the placental tissue [44]. Those findings question the hypothesis that the hypermethylation within the* HSD11B2* gene is responsible for the lower transcription efficiency and consequently for the lower expression of the enzyme in placenta. Furthermore, the experiment with isolated human placental cytotrophoblasts and* in vitro* differentiated syncytiotrophoblasts revealed that the tremendous increase in 11*β*-HSD2 expression during syncytialization is not a result of changes in the DNA methylation within the proximal region of* HSD11B2* promoter. The level of DNA methylation in the analyzed fragment of the* HSD11B2* gene was similar in cytotrophoblasts and syncytiotrophoblasts, suggesting another mechanism that is responsible for increase in the gene expression [45].

Lack of differences in the methylation level within* HSD11B2* in PE compared to normotensive pregnant women, combined with the reduced amount of mRNA in the preeclamptic subjects, further suggests that there may be other than the DNA methylation mechanism that would be responsible for the diminished enzyme expression [34]. Worth consideration are the phenomena related to the stability of the created mRNA because, which should be emphasized, even in case of efficient, undisturbed transcription, the formed mRNA does not ensure the efficient translation and complete expression of the protein. The mRNA molecules, the subjects for protein synthesis, can be degraded or the translation machinery can be blocked. The specific small molecules of the noncoding RNA, so-called microRNA, can act in both mentioned directions. The particular microRNAs can bind to the 3′-UTRs of mRNA of the target genes resulting in mRNA degradation and reduction of its half-life. They can also influence directly the translation machinery and block the process. First report in the field suggested that the expression of 11*β*-HSD2 is regulated by the action of specific microRNAs [46]. Up to now there is no information about the effect of microRNAs on the placental 11*β*-HSD2 expression and activity. It has been shown, however, that the microRNAs profiles in the placentas from preeclamptic pregnancies are significantly different from those obtained from normal pregnancies. The placental levels of certain microRNA molecules were found to be increased in pregnancies complicated by PE [47, 48].

The mRNA stability of 11*β*-HSD2 in the placenta is influenced by microRNAs, but it is also regulated by the mitogen-activated kinase p38 (p38 MAPK), which is necessary for fetal organogenesis and trophoblast growth and its invasion. The activation of p38 MAPK, triggered, for example, by cAMP pathway, was reported to go along with the increase of the amount of placental 11*β*-HSD2. It was suggested that p38 MAPK is involved in both basal and cAMP-induced expressions of* HSD11B2* [49, 50]. The fact that p38 MAPK is highly nitrated in preeclamptic placentas, which results in its lower catalytic activity, may partially explain the lower expression of 11*β*-HSD2 over the course of PE [51].

Modification of histone proteins is the third mechanism of epigenetic control of gene expression. Its role in the overall regulation of the expression of* HSD11B2* was suggested to be less important than the two other epigenetic mechanisms [37], but it seems that we only know less in this field. Histone acetylation is generally associated with the activation of transcription process, while histone methylation is known to be associated with transcriptional repression [45]. Baserga et al. described the changed structure of the chromatin of renal* HSD11B2* in rat fetuses with IUGR [36]. Moreover, the mentioned experiment [45] with human placental cytotrophoblasts and syncytiotrophoblasts showed that histone modifications may play an important role as mediators of cAMP-induced transcription of* HSD11B2*. To explain it properly, the role of cAMP pathway in* HSD11B2* expression should be, however, described. Shortly, the activation of cAMP pathway increases expression of the transcriptional factor Sp1 (regulating both basal and cAMP-induced* HSD11B2* expressions) and its binding to the special sites of the* HSD11B2* promoter, which correlates positively with the expression of* HSD11B2* in human placenta. Referring to the acetylation process, it was found that enhanced* HSD11B2* transcription, resulting from the activation of the cAMP signaling pathway, was also accompanied by the increased acetylation and decreased methylation of histone 3 lysine 9—H3K9 (associated with the* HSD11B2* promoter) [45, 50]. The crucial role in the process of H3K9 acetylation and consequently in the activation of* HSD11B2* transcription was attributed to the p300 protein—the transcriptional coactivator of Sp1 [52].

The* in vitro* studies revealed that the mRNA expression, the protein amount, and the activity of placental 11*β*-HSD2 are diminished in hypoxia [12, 53]. In hypoxic conditions, the enzyme expression is initially blocked at the level of translation (firstly, the downregulation of protein synthesis can be found), while later the transcription process is also affected (lower mRNA levels can be observed) [53]. Hypoxia in the placenta was reported as a consequence of the abnormal process of the trophoblast invasion at the early stages of pregnancy, and this phenomenon is in turn suggested as one of the mechanisms of PE development [6]. Therefore, according to this relationship, the impaired function of placental 11*β*-HSD2 observed over the course of PE would be the consequence and not the cause of PE. Moreover, it was reported that the reduced activity and expression of placental 11*β*-HSD2 observed in IUGR are not a result of the abnormalities in the structure of trophoblast cells, but they are rather due to the external factors (such as hypoxia) that are associated with IUGR [53]. The fact that this mechanism described for IUGR is true also for PE cannot be excluded. So the question whether the reduced activity of 11*β*-HSD2 is a cause or a consequence of PE remains open.

### 4.3. Substances Affecting Expression and Activity of the 11*β*-HSD2

The results of* in vitro* studies indicated several factors that may decrease the activity of 11*β*-HSD2 as well as the protein formation. Those included the locally elevated angiotensin II concentration or the increased availability of proinflammatory cytokines (interleukins Il-1*β* and Il-6 and tumor necrosis factor- (TNF-) *α*) [8, 54]. On the opposite, Xu et al. showed one of the endogenous anti-inflammatory autacoids, so-called lipoxin A4, to upregulate 11*β*-HSD2. Interestingly, the levels of this agent were significantly reduced in serum as well as in the placenta of PE patients as compared to healthy pregnant women and the phenomenon corresponded to lower placental 11*β*-HSD2 mRNA and protein amount [55]. Furthermore, the decreased expression of placental 11*β*-HSD2 was associated with the increased expression of peroxisome proliferator-activated receptor *α* (PPAR*α*) and the decreased expression of PPAR*γ* and this mechanism involves the effect on the transcription factor Sp1 [56].

Unfortunately, little is still known about the effect of various xenobiotics, used drugs, or lifestyle on the placental 11*β*-HSD2 function [5]. Yang et al. reported that the expression and the activity of the enzyme (assessed in human trophoblast cells) are reduced in the presence of cadmium, a commonly known pollutant, and the inhibitory mechanism includes the suppression of* HSD11B2* transcription [57]. The activity and expression of placental 11*β*-HSD2 are also reduced in the presence of caffeine and its primary metabolite—paraxanthine—and the effect is mediated by antagonizing adenosine A_2B_ receptor and thus is caused by decreasing intracellular cAMP level [58]. Triclosan, an agent widely used in hygienic products, was found to decrease the expression of 11*β*-HSD2 via the apoptosis induction in human placental syncytiotrophoblasts [59]. Furthermore, the expression of placental 11*β*-HSD2 in rats was found to be affected by the excessive folic acid supplementation, but the effect, achieved by the epigenetic modification in the* HSD11B2* gene, depended on the fetal gender [60]. The results of* in vitro* studies indicate that the chemicals commonly used in industry (phthalates, organotins, and perfluorinated compounds) [61], including food sector (butylated hydroxyanisole used for food preservation) [62], and in agriculture (dithiocarbamates and ziram) [63, 64] as well as the known mycotoxin—zearalenone [65]—have significant abilities to inhibit the 11*β*-HSD2. Moreover, naringenin, one of the active substances found in grapefruit juice, inhibits the enzyme function* in vitro*, but it is not clear whether the compound achieves* in vivo* concentrations that are large enough to significantly affect the 11*β*-HSD2 function [66]. However, the above-mentioned phenomena were not confirmed in humans, so their potential role in PE development remains unclear, which opens the field for future research.

Glycyrrhetinic acid should be definitely mentioned with relation to the 11*β*-HSD2 inhibitors. It is a natural substance, present in liquorice root, and it was found to induce the mild acquired form of AME [9]. Taking this fact into consideration, women that are genetically predisposed to pregnancy-specific hypertension should be very careful or even should avoid the liquorice consumption. The case of an 18-year-old nulliparous woman with a strong family history of PE who developed the severe early form of PE was described and in this case the significant liquorice consumption was suggested as the most probable cause of observed complications of pregnancy [67].

Studies on animal models have shown that the function of 11*β*-HSD2 is slightly blocked by some drugs widely used in the hypertension treatment, for example, methyldopa, dihydralazine, and furosemide [33, 68]. Furthermore, progesterone is a well-known potent inhibitor of 11*β*-HSD2 and its levels increase significantly during gestation [69]. Up to now, it has been reported, however, that progesterone concentrations observed over the course of PE are unchanged [26] or lower than in healthy pregnant women (not higher as could be expected considering the reduced function of 11*β*-HSD2 in PE) [70].

The activity of placental 11*β*-HSD2 depends also on the compounds secreted by the placenta and the decidua. Apart from the already mentioned progesterone, there is much interest in the human chorionic gonadotropin (hCG) [27].* In vitro* studies showed that hCG influences the expression of placental 11*β*-HSD2 via the interference of, discussed in earlier sections, cAMP and p38 MAPK pathways [50]. Moreover, both the expression and the secretion of hCG are increased by F, a hormone which as a substrate for 11*β*-HSD2 enhances also the expression of 11*β*-HSD2. Thus, the upregulatory effect of F on 11*β*-HSD2 expression may be partly mediated by hCG [71]. After the 20th week of gestation, the levels of serum hCG in pregnancies complicated by PE are consistently higher than in healthy pregnant women [72]. It must be stated, however, that the precise role of hCG in pathogenesis of PE is still unknown.

## 5. Conclusions

The literature data indicate that the function of placental 11*β*-HSD2 in PE is reduced. This is evidenced by the diminished mRNA levels and the activity of the enzyme. Moreover, it is supported by the decreased apparent activity of the enzyme reflected in the increased F concentrations in cord blood and higher amount of F in the placenta in PE when compared to normal pregnancies. These data are, however, mostly descriptive, and the mechanism of these abnormalities remains unclear which opens the field for future research. However, the complexity of the issue has to be considered while various phenomena can be involved at any stage of protein synthesis. Those phenomena can include the DNA sequence alterations and the epigenetic changes (such as hypermethylation and impact of microRNA on mRNA stability), but also the influence of inhibitors (e.g., progesterone or its metabolites). The reduced placental 11*β*-HSD2 activity may, in turn, result in the overexposure of the child to F and, in consequence, it can lead to lower birth weight or even to IUGR. These, according to “fetal programming,” affect adversely not only the perinatal outcomes, but also the later life of the child.
